# *Ex Vivo* Activity of Cardiac Glycosides in Acute Leukaemia

**DOI:** 10.1371/journal.pone.0015718

**Published:** 2011-01-05

**Authors:** Helene Hallböök, Jenny Felth, Anna Eriksson, Mårten Fryknäs, Lars Bohlin, Rolf Larsson, Joachim Gullbo

**Affiliations:** 1 Department of Haematology, University Hospital, Uppsala University, Uppsala, Sweden; 2 Department of Medicinal Chemistry, Division of Pharmacognosy, Uppsala University, Biomedical Centre, Uppsala, Sweden; 3 Department of Medical Sciences, Division of Clinical Pharmacology, Uppsala University Hospital, Uppsala, Sweden; Florida International University, United States of America

## Abstract

**Background:**

Despite years of interest in the anti-cancerous effects of cardiac glycosides (CGs), and numerous studies *in vitro* and in animals, it has not yet been possible to utilize this potential clinically. Reports have demonstrated promising *in vitro* effects on different targets as well as a possible therapeutic index/selectivity *in vitro* and in experimental animals. Recently, however, general inhibition of protein synthesis was suggested as the main mechanism of the anti-cancerous effects of CGs. In addition, evidence of species differences of a magnitude sufficient to explain the results of many studies called for reconsideration of earlier results.

**Principal Findings:**

In this report we identified primary B-precursor and T-ALL cells as being particularly susceptible to the cytotoxic effects of CGs. Digitoxin appeared most potent and IC_50_ values for several patient samples were at concentrations that may be achieved in the clinic. Significant protein synthesis inhibition at concentrations corresponding to IC_50_ was demonstrated in colorectal tumour cell lines moderately resistant to the cytotoxic effects of digoxin and digitoxin, but not in highly sensitive leukaemia cell lines.

**Conclusion:**

It is suggested that further investigation regarding CGs may be focused on diagnoses like T- and B-precursor ALL.

## Introduction

Cardiac glycosides (CGs) are a group of plant-derived compounds that have been used for many years in traditional medicine and that are currently used in treatment of cardiac failure and atrial fibrillation. In parallel to this use, CGs have also received attention as potential drugs in the treatment of various malignant diseases. Epidemiological observations suggest that patients on digitalis medication diagnosed with breast cancer in general present with lower proliferating tumours of smaller size, and subsequently better prognosis than control groups [1], [2], [3], [4], and that a high concentration of digitoxin could reduce the risk of developing leukaemia, lymphoma or urogenital cancer [5]. *In vitro* experiments have shown that CGs can induce cell death in several cell lines derived from solid cancers [6], [7] as well as in leukaemic cell lines [8], [9], [10], [11].

In the myocardium, CGs bind reversibly to the α-subunit of Na^+^/K^+^ ATPase, leading to a rise in intracellular sodium levels, which then results in an increase of calcium ions in the myocytes. The mechanism of the cytotoxic effects of CGs on tumour cells has been a subject of many studies but it largely remains unanswered. Binding to Na^+^/K^+^ ATPase is not only a way of regulating ion pumps in the cell membrane but it can also activate several signalling pathways in the cell. For example, calcium-dependent activation of caspases and other hydrolytic enzymes [7], [12], [13], generation of reactive oxygen species (ROS) [14], topoisomerase inhibition [15], interference with signal transduction pathways (e.g. Src-mediated phosphorylation of epidermal growth factor receptor (EGFR) and induction of the cell cycle inhibitor p21*^Cip1^* [16] have all been associated with the anti-tumour effects of CGs.

Digoxin-Like Immunoreactive Factors (DLIFs, also termed Digitalis-Like Compounds (DLCs)) are endogenous steroids identified in human tissues and they are identical or similar to plant and amphibian steroids. The DLIFs are believed to be synthesized in the adrenal gland, and to affect ion transport via Na^+^/K^+^ ATPase. Binding of these compounds to Na^+^/K^+^ ATPase may activate changes in intracellular Ca^2+^ homeostasis and in specific gene expression [17], and may be associated with the development of malignancies [18]. Interestingly, DLIFs selectively induce apoptosis in a human acute T-cell lymphoblastic leukaemia cell line but not in the myelogenous leukaemia cell line K-562 or healthy human peripheral blood mononuclear cells (PBMCs) [19].

Despite years of interest in these effects and numerous studies *in vitro* and in animals, it has not yet been possible to utilize the anti-cancerous potential of CGs clinically. Recently, a very discouraging report on this issue was published, suggesting general inhibition of protein synthesis as the main mechanism of the anti-cancerous effects of CGs, and species differences of a magnitude sufficient to explain the results of most preclinical studies [20].

During a routine screening programme carried out *in vitro* we observed that some samples of acute leukaemia were extremely sensitive to the cytotoxic effects of digitoxin, thus prompting further investigation. Hence this study was undertaken to categorize the activity of some CGs in primary cultures from patients with various leukaemic diagnoses, and to determine if general protein inhibition is the dominant mechanism of action, and if a therapeutic index *in vitro* exists.

## Materials and Methods

### Patient Samples and Cell Lines

Cryopreserved cells from bone marrow or peripheral blood from adult patients with B-precursor or T-acute lymphoblastic leukaemia (ALL), acute myeloid leukaemia (AML) and chronic lymphocytic leukaemia (CLL) were used in the study. Peripheral blood mononuclear cells (PBMCs) from healthy donors were used as controls.

Informed consent was obtained from all patients (verbal until 2006 and written thereafter) to save diagnostic samples in a biobank to be used for scientific research. The informed consent was verbal until 2006 (in accordance with the approval from the Ethics Committee) and the Ethics Committee did not demand that the consent should be documented at this time (until 2006). Since 2006 written consent has been obtained. Sampling for drug sensitivity testing was approved by the local Ethics Committee in Uppsala (Regionala etikprövningsnämnden i Uppsala, Sweden, approval number Dnr 21/93). The samples from the biobank used in the study were coded but labelled with diagnosis.

The T-lymphoblast-like cell line CEM/VBL_100_ (CCRF-CEM) [21] was kindly donated by professor W.T. Beck, St. Jude's Children's Research Hospital, USA and the B-precursor Philadelphia-positive cell line SUP-B15 [22] was obtained from Deutsche Sammlung von Mikroorganismen und Zellkulturen GmbH, Germany. The cells were grown in complete medium (CCRF-CEM in RPMI 1640 and SUP-B15 in McCoy's 5A) and split twice weekly. The colorectal adenocarcinoma cell lines Hct116, HT29 and CC20 were used as comparator cell lines as regards effects on protein synthesis.

### Chemicals and Reagents

The CGs digitoxin, digoxin and ouabain were purchased from Sigma (Sigma-Aldrich, Stockholm, Sweden). The compounds were dissolved in DMSO and further dilution was in PBS. The drugs were tested at five concentrations (ten-fold dilution steps) ranging from 100 µM to 0.01 µM or 10 µM to 0.001 µM. The maximum concentration of DMSO did not exceed 1% in the cell cultures. The serially diluted stock solutions were transferred to 384-well microtitre plates (NUNC, Roskilde, Denmark) and control wells were filled with PBS only (5 µl/well).

### Measurement of Cytotoxic Activity

The cytotoxic activity of the CGs was measured by using a fluorometric microculture cytotoxicity assay (FMCA) as previously described [23], [24]. The method is based on measurement of the fluorescence derived by hydrolysis of fluorescein diacetate (FDA) to fluorescein by cells with intact plasma membranes. Cell suspensions were seeded into drug-prepared 384-well microtitre plates. Wells with medium only served as blanks. The plates were incubated at 37°C for 72 hours; thereafter FMCAs were performed using an automated Optimized Robot for Chemical Analysis (Orca; Beckman Coulter Fullerton, CA) programmed through SAMI software (Beckman Coulter). The plates were washed in physiological buffer and FDA added. After 50 min of incubation at 37°C, fluorescence was measured at 485/520 nm using a Fluostar Optima microplate reader (BMG Technologies, Germany). The fluorescence measured is proportional to the number of living cells in each well.

Because a 30–40 nM plasma concentration of digitoxin can be maintained in patients for many days, additional experiments with extended incubation time (6 days) was performed. Leukaemic patient cells and the CCRF-CEM cell line was treated with therapeutically achievable concentrations (10, 30 and 50 nM) of digitoxin in 96-well microtiter plates. To simulate a situation with daily dosing, 50% of the medium (including drug to treated cells) was exchanged every day.

Quality control was evaluated for each test by demanding a signal/blank ratio of >10 and a coefficient of variation in controls and blanks of <30%. The proportion of leukaemic cells in primary cultures should exceed 70% on days zero or three when examined morphologically.

### Measurements of protein and nucleic acid synthesis inhibition

Effects on DNA and protein synthesis were monitored in Cytostar-T® plates (available in the “*In Situ* mRNA Cytostar-T® assay” kit, Amersham International, Buckinghamshire, UK) using ^14^C-labelled thymidine and leucine. A Cytostar-T® plate is a 96-well microtitre plate with scintillants molded into the transparent polystyrene bottom. When labelled substrate is absorbed into the intracellular compartment of the cells at the bottom of the wells, the radioisotope is brought into proximity with the scintillant, thereby generating a detectable signal. Free radiolabelled substrate in the supernatant is unable to stimulate the scintillant [25], [26].

CCRF-CEM and SUP-B15 cells were suspended in fresh medium containing ^14^C-thymidine (111 nCi/ml; for DNA experiments) or ^14^C-leucine (222 nCi/ml; for protein experiments), yielding final radioactivity in the wells of 20 and 40 nCi, respectively. Cell suspension (50×10^3^ cells in 180 µl) was added to each well; blank wells had isotope-containing medium only. Drugs (digoxin and digitoxin at final concentrations of 10 µM to 1 nM) and PBS in test and control wells were added in duplicate (20 µl per well) 2 hours after cell seeding, when the measured radioactivity in cell-containing wells was at least double compared with blank wells. Radioactivity was measured with a computer-controlled Wallac 1450 MicroBeta® trilux liquid scintillation counter (Wallac OY., Turku, Finland) immediately after addition of the cell suspension and at different time points up to 72 hours. Between measurements, the plates were stored in an incubator at 37°C. During measurement, the plates were covered with a plate sealer to inhibit microbiological contamination.

## Results

The cytotoxic activities of digitoxin and ouabain were studied in primary leukaemic cells: T-ALL (n =  digitoxin 4; ouabain 7), B-precursor ALL (n = 10; 6), AML (n = 11;11), CLL (n = 9; 6) and PBMCs (n = 4; 8) using the FMCA. Similar tests on the activity of digoxin, digitoxin and ouabain were performed in the leukaemia cell lines CCRF-CEM and SUP-B15. All tests were carried out in triplicate.

**Figure 1:**
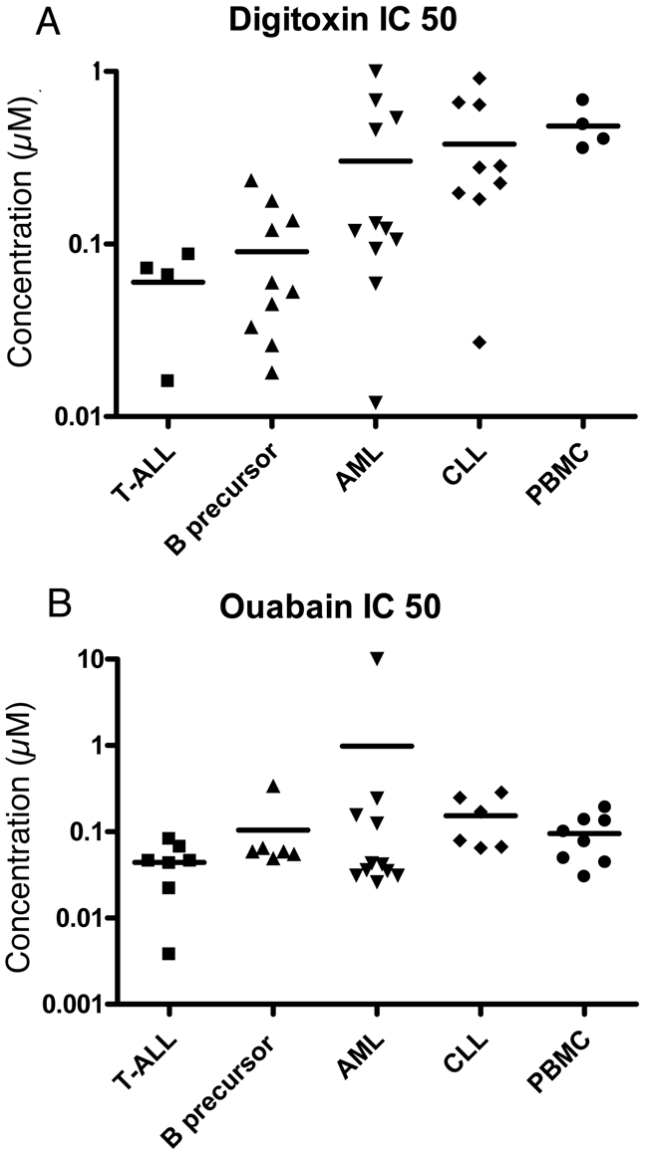
Cytotoxic IC_50_ values. Cytotoxic IC_50_ values (µM) for digitoxin (A) and oubain (B) in primary cultures of human leukaemia cells (T-ALL, B-precursor ALL, AML and CLL) and PBMCs.

The primary T- and B-precursor ALL cells were significantly more sensitive to digitoxin than CLL cells (Mann–Whitney test, p = 0.02 and 0.006 respectively) and PBMCs (p = 0.02 and 0.005 respectively) (Figure 1A). The median IC_50_ value regarding digitoxin was 0.07 µM for T-ALL and 0.06 µM for B-precursor ALL cells, compared with 0.44 µM for PBMCs (Table 1). For the AML cells a scattered distribution regarding IC_50_ values was observed. As regards ouabain, the IC_50_ value was significantly lower (Mann–Whitney test, p = 0.02) for the T-ALL cells than for CLL cells but otherwise no significant differences were observed in the different leukaemic cells or the PBMCs (Figure 1B and Table 1).

**Table 1:** IC_50_ (µM) regarding the effects of digitoxin and ouabain on primary leukaemic cells and PBMCs.

	IC 50%	T-ALL	B-precursor ALL	AML	CLL	PBMCs
Digitoxin	Median (range)	0.07 (0.02–0.09)	0.06 (0.02–0.23)	0.12 (0.01–1)	0.28 (0.03–0.92)	0.44 (0.36–0.68)
Ouabain	Median (range)	0.05 (0.004–0.08)	0.06 (0.05–0.34)	0.04 (0.03–10 *)	0.12 (0.06–0.28)	0.09 (0.03–0.19)

*Never reached IC 50%.

**Figure 2:**
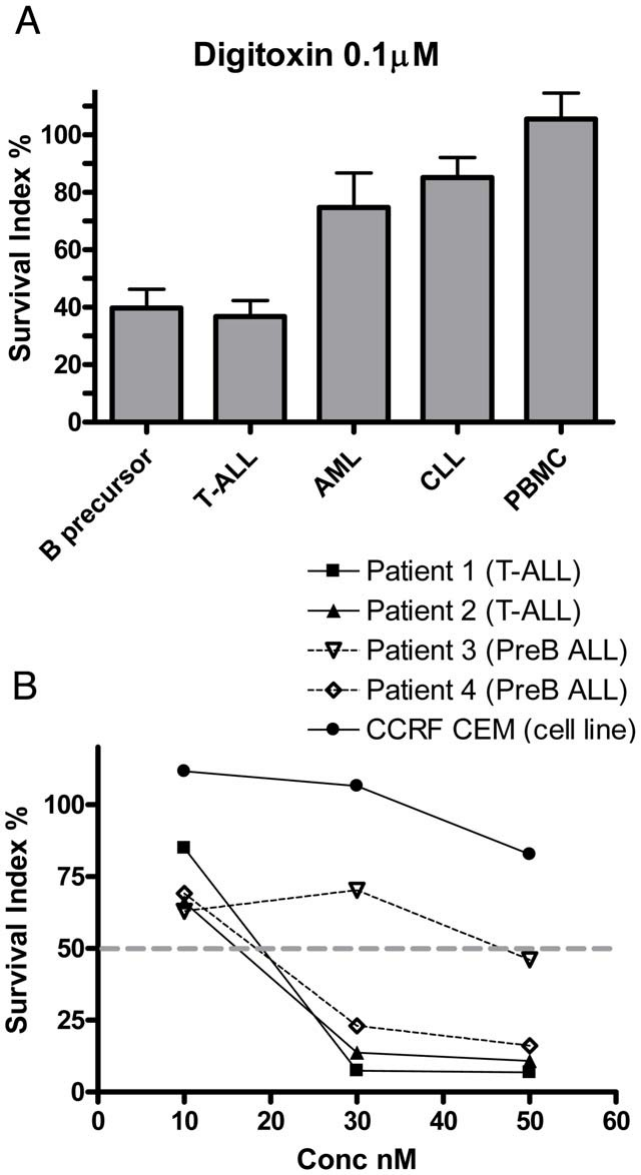
Cytotoxic effects of digitoxin. A: Cytotoxic effects of digitoxin (0.1 µM) expressed as Survival Index % (*vs.* untreated control cells) in the various leukaemia types (T-ALL, B precursor ALL, AML and CLL) and PBMC using the standard 72 h assay. B: Effects of therapeutically achievable digitoxin concentrations against leukaemic cells from patients and CCRF-CEM cell line over six days with daily medium change.

At 0.1 µM the B-precursor and T-ALL cells were significantly more sensitive to digitoxin than the CLL cells and PBMCs (Mann–Whitney test, B-precursor ALL *vs.* CLL p = 0.0003, B-precursor ALL *vs.* PBMCs p = 0.002, T-ALL *vs.* CLL, p = 0.004 and T-ALL *vs.* PBMCs, p = 0.03). In addition, the B-precursor ALL cells were more sensitive than the AML cells (p = 0.02) (Figure 2A). With extended exposure (6 days) both T- and B-precursor ALL cells appeared sensitive at clinically achievable concentrations (Figure 2B).

The cell line SUP-B15 was highly sensitive to all tested CGs, with IC_50_ values as follows: digitoxin 0.002 µM, ouabain 0.004 µM and digoxin 0.03 µM. The T-lymphoblast-like cell line CCRF-CEM showed effects similar to those in the primary ALL cells, with IC_50_ values of 0.04 µM for ouabain, 0.12 µM for digitoxin and 0.22 µM for digoxin. In both cell lines there was a tendency towards a lower sensitivity to digoxin than to the other two CGs.

**Figure 3:**
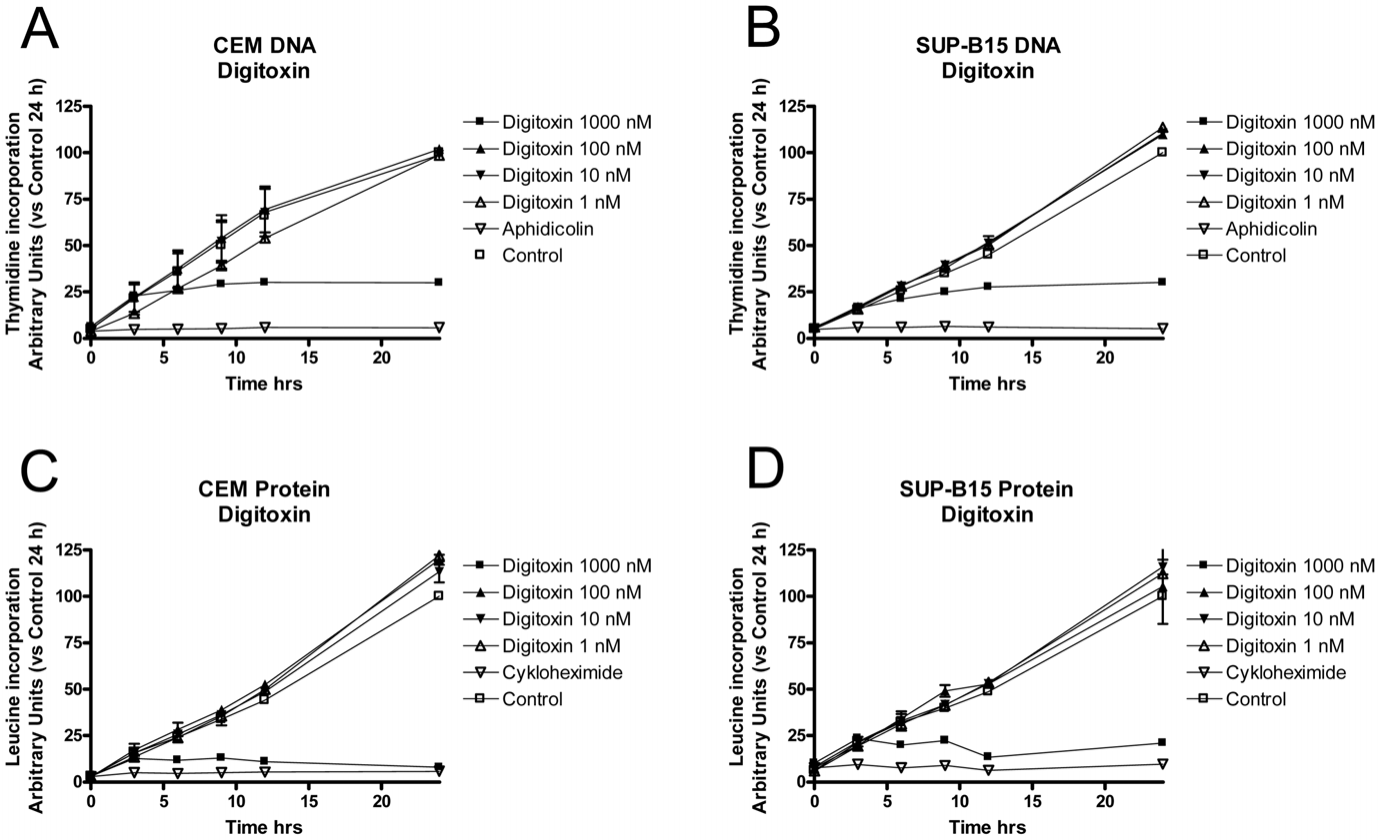
Effects of digitoxin on DNA synthesis in leukaemic cell lines. Effects of digitoxin exposure on DNA synthesis (i.e. thymidine incorporation) in CCRF-CEM (A) and SUP-B15 (B) cells. The DNA polymerase inhibitor aphidicolin (15 µM) was used as a positive control. Effects of digitoxin exposure on protein synthesis (i.e. leucine incorporation) in CCRF-CEM (C) and SUP-B15 (D) cells. The ribosomal inhibitor cycloheximide (36 µM) was used as a positive control.

**Figure 4:**
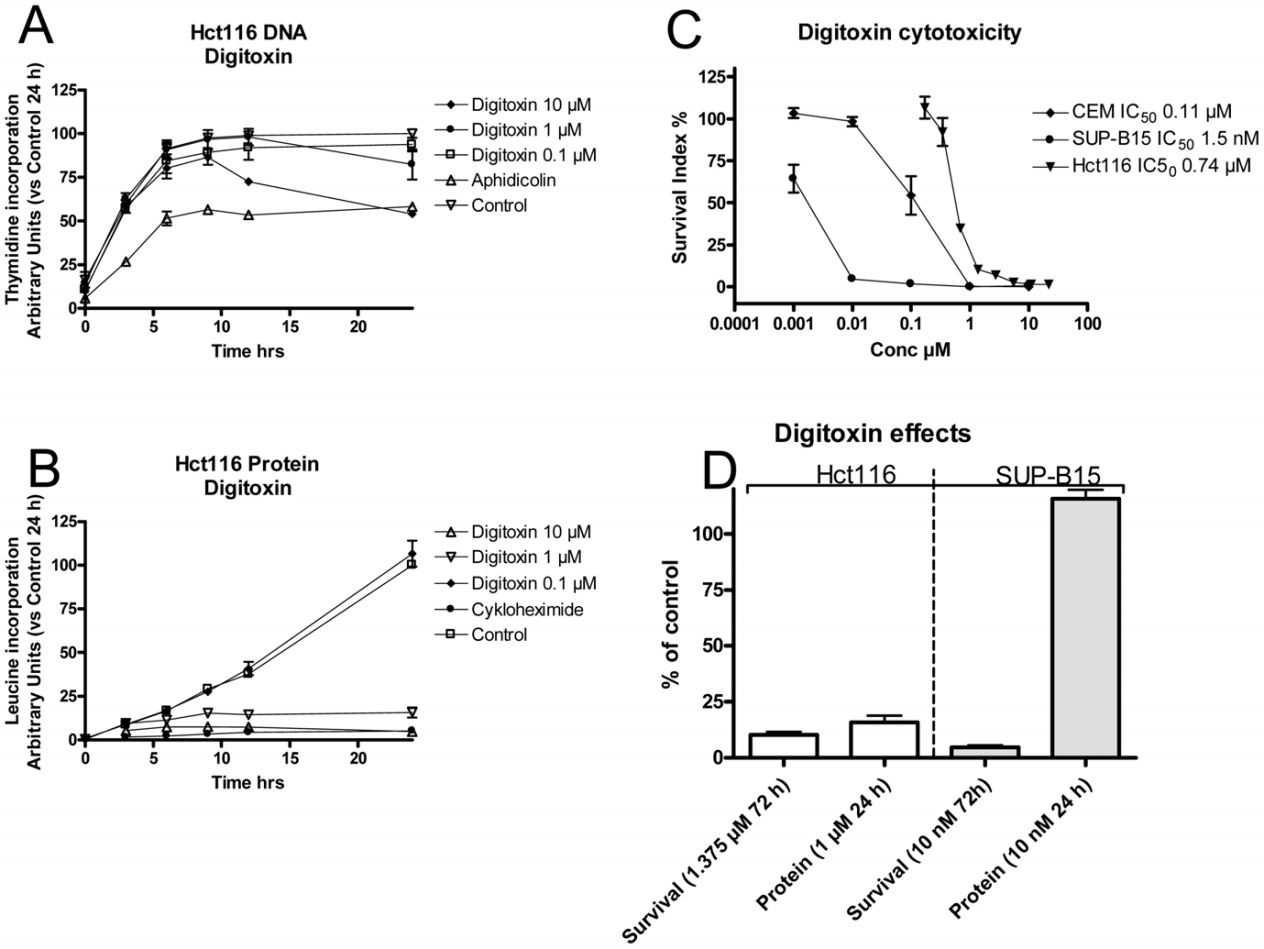
Comparison between colorectal and leukaemic cell lines. For comparative purposes the effects of digitoxin on DNA and protein synthesis (A and B respectively) were also monitored in the colorectal adenocarcinoma cell line Hct116. Figure 4C shows differences in cytotoxic activity of digitoxin in the Hct116 cell line and the leukaemic cell lines (CCRF-CEM and SUP-B15) used in the present study. In Figure 4D the effects on survival (at 72 h, measured by FMCA) and protein synthesis at 24 hours at digitoxin concentrations slightly exceeding the IC_50_ values in HT29 and SUP-B15 cells are shown.

Both digoxin and digitoxin inhibited DNA as well as protein synthesis in CCRF-CEM and SUP-B15 cells. The effects of the specific inhibitors aphidicolin and cycloheximide, used as positive controls, were strong and immediate. As presented in Figure 3 (digitoxin only; digoxin showed similar results), the effects were, however, only observed at relatively high concentrations, and at 100 nM, surprisingly, no significant effects were detected during the 24-h observation period. At the highest concentration tested (1.0 µM), well exceeding the IC_50_ value for cytotoxicity, the effects on DNA and protein synthesis were similar in time and magnitude, i.e. the cells tended to “shut down” in an expected manner due to the toxic insult, presumably related to severe ionic imbalance. To put these results into perspective, a comparator cell line, the adenocarcinoma cell line Hct116was also analysed. As the Hct116 cell line is more tolerant to the cytotoxic effects of glycosides, slightly higher CG concentrations were used. Figure 4 shows the effects of various digitoxin concentrations on DNA (A) and protein synthesis (B). In contrast to the results in the leukaemia cell lines, protein synthesis in Hct116 cells was decreased more efficiently (i.e. at concentrations corresponding to cytotoxic activity) and at an earlier time point than DNA synthesis. With 1 µM digitoxin, protein synthesis in Hct116 cells was effectively inhibited at early time points (significant from 6 hours), while DNA synthesis did not appear to slow down until 24 h. This concentration is comparable to the cytotoxic IC_50_ value for Hct116 cells measured in the FMCA (0.71 µM, Figure 4C). The effects in two other colorectal adenocarcinoma cell lines (HT29 and CC20) were similar (not shown). The leukaemic SUP-B15 cell line was approximately 500 times more sensitive to the cytotoxic effects of digitoxin, with an IC_50_ value of 1.5 nM (Figure 4C). Despite this, exposure of SUP-B15 cells to 10 nM digitoxin had no effect on protein synthesis up to 24 h (Figures 3D and 4D), but it effectively reduced viability at 72 h (Figure 4D). Results in the leukaemic CCRF-CEM cell line were similar (not shown).

## Discussion

In this study we have identified primary B-precursor and T-ALL cells as being particularly susceptible to the cytotoxic effects of CGs, being significantly more sensitive than CLL cells and PBMCs. Digitoxin was the most potent of the CGs tested, and the measured IC_50_ value was comparable with therapeutic serum concentrations, especially if exposure time was extended to 6 days. The therapeutic concentration range for CGs in clinical use is narrow – for digitoxin 15–40 nmol/l (10–30 ng/ml) [27]. This, of course, raises the question of whether or not there could be a place for CGs in the treatment of acute leukaemia, despite negative results in solid tumours, for example. In ALL maintenance treatment with moderate doses of 6-mercaptopurin and methotrexate for a period of approximately two years has a documented effect on the risk of relapse [28]. The intensity of the treatment is limited by haematological and/or liver toxicity. New drugs with different toxicity spectra could clearly be of substantial benefit in this setting. Therefore, digitoxin as an addition to conventional maintenance chemotherapy in ALL could be proposed as a future clinical study, but only after careful preclinical investigation of possible drug-drug interactions. For example, previous studies have indicated that digitoxin at concentrations commonly found in the plasma of cardiac patients, significantly reduced etoposide and idarubicin-induced topoisomerase II cleavable complexes in K562 leukemia cells [29].

The cytotoxic effect of digitoxin on both primary ALL cells as well as in the extremely sensitive cell line SUP-B15S indicates that a mechanism of inducing cell death other than inhibition of sodium- and potassium-activated adenosine triphosphatase (Na^+^/K^+^ ATPase) may be possible. Numerous effects of cardiac glycosides in cancer cell lines ultimately leading to apoptosis have been demonstrated previously. Most of these effects are probably mediated through the main target enzyme [30]. However, interpretation of experiments involving established cell lines calls for caution because of possible genetic drift and altered properties. This is illustrated by the extreme sensitivity towards CGs in the cell line SUP-B15, while results in the CEM cell line were similar to those in the primary leukaemic cells.

Despite years of interest in these effects and numerous studies *in vitro* and in animals, it has not yet been possible to utilize the anti-cancer potential of CGs clinically. Several preclinical studies have demonstrated these compounds to be involved in selective control of human proliferation, which support the use of CGs to treat malignancies [30], [31]. Different glycosides with different profile regarding cellular effects and cardiotoxicity have been developed, and several clinical trials have been performed. For example, UNBS-1450, a semisynthetic cardenolide, with good preclinical activity against NSCLC has entered a phase I clinical trial in Belgium [32]. A phase I trial of Anvirzel™, an aqueous extract from *Nerium Oleander*, in patients with refractory solid tumors has been reported [33]. Studies of the addition of digoxin to combination chemotherapy and immunotherapy in patients with advanced malignant melanoma have also been initiated [34], the study has not been finally reported.

There are several possible explanations for this slow progression into clinical practise; one is of course the possibility of *in vitro* biases, and/or species differences. For example, it has been demonstrated that oleandrin activates MAPK and JNK and also induces expression of FasL, leading to apoptosis in human, but not in murine cells (28). This difference has also been detected at the cellular membrane level, as oleandrin altered its fluidity, inhibiting Na^+^/K^+^ ATPase activity, and increasing intracellular free Ca^2+^ levels, followed by calcineurin activity only in human, but not in murine cells. The results suggested that murine plasma membranes might be different from human membranes, which interact with oleandrin, disturbing the Na^+^/K^+^ ATPase pump and resulting in calcification, followed by induction of Ca^2+^-dependent cellular responses such as apoptosis [35]. In line with these findings, Perne *et al.* recently published experimental results suggesting that general inhibition of protein synthesis is the main mechanism of the anti-cancerous effects of CGs in human cells, and that physiological species differences may explain the previously observed sensitivity of human cancer cells in mouse xenograft experiments [20]. It was proposed that inhibition of protein synthesis is directly related to effects on the Na^+^/K^+^ ATPase pump. Indeed, this protein synthesis-inhibiting mechanism is supported by observations in the colorectal adenocarcinoma cell line Hct116 (Figure 4). With 1 µM digitoxin, protein synthesis is effectively inhibited at early time points (significant from 6 hours), leading to decreased cell numbers at 72 hours. It might be concluded that Hct116 cells die because of an inability to synthesize vital proteins. The results from the leukaemia cell lines appear to be in sharp contrast to this – protein synthesis levels at time points up to 24 h are unaffected by concentrations having severe effects on cellular viability. Thus, it may be concluded that these cells stop synthesizing their proteins because they are dying, rather than the opposite.

### Conclusions

Cardiac glycosides have been used for treatment of congestive heart failure and atrial fibrillation for over a century. During the last twenty years this class of compounds (e.g. digitoxin and oubain) has received much attention as regards their potential as anti-cancer agents, based on epidemiological as well as experimental findings. Despite great efforts the mechanism behind the anti-cancerous effects has been difficult to determine, as have the potential benefits in the clinic. The recently published study by Perne *et al.* [20] shed some light on this issue, but also strongly discouraged further clinical development of CGs, and derivatives thereof, for use in this therapeutic area. In this study we describe how some subsets of leukaemic cells from patients express high sensitivity towards CGs, particularly digitoxin, and at concentrations that may be achieved in the clinic. Furthermore, it was shown that the suggested mechanism of protein inhibition is valid in tumour cell lines with moderate or high resistance to the cytotoxic effects of CGs, but probably not in highly sensitive cancer cell lines. It is thus suggested that further investigation may be focused on diagnoses like T- and B-precursor ALL.
